# Role of the DNA-Binding Protein pA104R in ASFV Genome Packaging and as a Novel Target for Vaccine and Drug Development

**DOI:** 10.3390/vaccines8040585

**Published:** 2020-10-03

**Authors:** Ana Catarina Urbano, Fernando Ferreira

**Affiliations:** CIISA—Centro de Investigação Interdisciplinar em Sanidade Animal, Faculdade de Medicina Veterinária, Universidade de Lisboa, Avenida da Universidade Técnica, 1300-477 Lisboa, Portugal; acurbano@fmv.ulisboa.pt

**Keywords:** African swine fever, ASFV, genome packaging, viral DNA-packaging proteins, NCLDV, vaccine, antiviral therapy, stilbenes, pA104R

## Abstract

The recent incursions of African swine fever (ASF), a severe, highly contagious, transboundary viral disease that affects members of the Suidae family, in Europe and China have had a catastrophic impact on trade and pig production, with serious implications for global food security. Despite efforts made over past decades, there is no vaccine or treatment available for preventing and controlling the ASF virus (ASFV) infection, and there is an urgent need to develop novel strategies. Genome condensation and packaging are essential processes in the life cycle of viruses. The involvement of viral DNA-binding proteins in the regulation of virulence genes, transcription, DNA replication, and repair make them significant targets. pA104R is a highly conserved HU/IHF-like DNA-packaging protein identified in the ASFV nucleoid that appears to be profoundly involved in the spatial organization and packaging of the ASFV genome. Here, we briefly review the components of the ASFV packaging machinery, the structure, function, and phylogeny of pA104R, and its potential as a target for vaccine and drug development.

## 1. Introduction

African swine fever (ASF) is a severe hemorrhagic viral disease that affects members of the *Suidae* family, including domestic pigs, wild boar, and warthogs (*Phacochoerus africanus*), considered the original vertebrate host. It can also involve soft tick vectors of the genus *Ornithodoros*, which have played an important role in the epidemiology of the disease in the Iberian Peninsula and Africa [1]. ASF is caused by a large double-stranded DNA virus (ASFV), the sole member of the *Asfarviridae* family, genus *Asfivirus*, and the only known DNA arbovirus [2]. There is no approved vaccine or treatment available for ASF, and control of the disease depends on implementation of rigorous import policies and biosecurity measures, including strict control of animal and pork movements (especially informal ones), and early detection and culling of infected animals. Currently ASF is endemic in several sub-Saharan African countries and on the island of Sardinia (Italy) in Europe. Since 2016, a significant increase in the number of outbreaks was observed, the majority of which were in Europe, with many countries reporting the first occurrence of the disease (Moldova in September 2016, then Czech Republic in June 2017, followed by Romania in July 2017, Hungary in April 2018, Bulgaria in August 2018, Slovakia in July 2019, and most recently, Serbia in January 2020, Greece in February 2020 and Germany in September 2020). Southeast Asia, however, saw the highest impact in terms of animal losses, with the disease spreading uncontrollably from China (where it was first reported in August 2018) to 11 other countries (India, Indonesia, Democratic People’s Republic of Korea, Republic of Korea, Laos, Myanmar, Papua New Guinea, Philippines, Russia, Timor-Leste and Vietnam) [3,4]. Sequencing of several contemporary ASFV isolates suggests high similarity with the strain responsible for a 2007 outbreak in the Caucasus region of the Republic of Georgia which subsequently spread to neighboring countries (Armenia, Azerbaijan, and nine states of the Russian Federation) [5]. The general lack of biosecurity measures and inability to control outbreaks effectively, especially in the backyard sector, as well as the existence of large areas of interaction between free-ranging pigs and wild boar, means there is a high risk that ASF may become endemic in this region and spread further into unaffected areas [1,6]. In the absence of a vaccine, the ASF incursion into countries such as China which rely heavily on the pork production industry and own almost half the world’s domestic pig population, has had a catastrophic impact on trade and pig production, with serious implications for global food security.

## 2. ASFV Genome Replication and Packaging

### 2.1. Viral DNA-Packaging Proteins

In cellular organisms, DNA-packaging proteins bind DNA and promote its bending, organizing it into highly compacted structures (called chromatin) which have a central role in the regulation of gene expression. Evolutionary analysis has shown that the primary DNA-packaging proteins involved in the organization of chromatin are different across the three domains of life. In bacteria, the primary DNA-packaging proteins are members of the HU/IHF superfamily (also called Type II DNA-binding proteins—DNABII—or bacterial histone-like proteins, pfam PF00216) [7]. Conversely, most eukaryotes and several archaea contain histones, highly basic proteins that form the characteristic octameric DNA structural (and functional) unit termed the nucleosome [8]. 

Double-stranded DNA viruses display a large variety of proteins that interact with host chromatin whose distribution seems to be influenced mainly by viral genome size and the domain to which the host of the virus belongs [9]. Smaller viruses (e.g., Papoviruses in eukaryotes, Salterproviruses in archaea, and Tectiviruses in bacteria) usually possess a minimal DNA replication apparatus consisting of a few multifunctional proteins that mediate several distinct interactions with host chromatin proteins and viral or host DNA [9]. Larger viruses such as the animal Adenoviruses and Herpesviruses, archaeal Lipothrixviruses and Baculoviruses, and several lineages of caudate bacteriophages possess distinctive virus-specific DNA-binding and/or adaptor domains (e.g., RRM-like domain in E2 from Papillomaviruses and LANA/EBNA1 from vertebrate Herpesviruses), and additionally encode several enzymes which catalyze chromatin status and chromosomal architecture (e.g., SWI2/SNF2 P-loop ATPases from archaeal Lipothrixviruses and several bacteriophages), and covalently modify chromatin components (e.g., SET domain histone methyltransferase from *Paramecium bursaria Chlorella virus*) [9].

The largest DNA-viruses (over 150 kb and extending up to 2.5 Mbp (*Pandoravirus salinus*) [10]) typically encode hundreds to thousands of proteins. Eukaryotic viruses in this range include Polydnaviruses such as the wasp *Cotesia congregata Bracovirus* [11], the recently designated *Cyprinid Herpesvirus 3* (CyHV-3) which infects carp [12], and the large nucleocytoplasmic DNA virus (NCLDV) clade which consists of seven distinct families of eukaryotic dsDNA viruses, namely *Phycodnaviridae*, *Poxviridae*, *Asfarviridae* (ASFV), *Asco*- and *Iridoviridae*, *Mimiviridae*, *Marseilleviridae* [13], and the proposed novel *Medusaviridae* [14]. Mimivirus and many other NCLDV subfamilies have evolved a unique genome packaging mechanism that is comparable to chromosome segregation in bacteria and archaea [15,16] and requires a number of specific enzymes, such as packaging ATPases, recombinases, DNA polymerases, helicases, and topoisomerases, as well as histones or histone-like proteins [17]. Mimiviruses package their genome into preformed procapsids through a nonvertex portal driven by the vaccinia virus A32-type virion packaging ATPase [12], homologous to the bacterial FtsK/HerA family prokaryotic chromosome segregation and packaging motors [15,16], and use similar revolving mechanism for genome packaging [18]. The ATPase interacts with other genome packaging components such as recombinase/s, a type II topoisomerase, and possibly several as yet unidentified components to form a complex that is competent for both resolving the genomes into unit lengths and translocating them into empty capsids [15,16]. In Vaccinia virus (*Poxviridae*) genome packaging is slightly different. The packaging ATPase complex collaborates with host type II topoisomerase for decatenation and genome replication after which the packaging ATPase assembly docks a copy of the genome onto the capsid vertex for packaging and leaves [16]. Although the mechanisms of assembly and genome encapsidation in ASFV have not been fully characterized, the similarities in genome structure with Poxviruses and the presence of replication intermediates consisting of head to head genome concatemers suggests they may share a similar replication model [19]. Further, data from electron microscopy indicates that in ASFV the viral DNA begins to condense into a pronucleoid and is then inserted, at a single vertex, into an empty particle which then goes through an intermediate phase of consolidation to produce the characteristic mature virions [19]. Thus, the overall composition of the ASFV packaging machinery is probably similar to Mimivirus and other NCLDVs.

### 2.2. ASFV Genome Structure

ASFV genomic organization also resembles that of other NCLDVs. The ASFV genome is a single molecule of linear double stranded DNA organized in a central relatively conserved and evolutionary stable region of about 125 kbp capped by two variable regions with a length of 38–47 kbp for the left, and 13–16 kbp for the right DNA ends [20]. Each DNA strand is covalently closed at both ends by a 37 nt hairpin loop followed by terminal inverted repeats of 2.1 kbp, which are characterized by numerous tandem repeat arrays. When examined in opposite polarities the AT-rich hairpin loops are inverted and complementary [21]. The genome varies in length between 170 and 190 kbp and encodes between 151 and 167 open reading frames (ORF) spaced closely along both chains of the viral DNA and separated by short intergenic regions. About half of them lack any known or probable function [22,23,24]. Differences in genome length mainly result from deletions or additions of up to 8.6 kbp in the left- and right-hand variable regions, with gain or loss of members of different multigene families (MGFs) [20,22]. Interestingly, MGFs do not share similarity to other known NCLDV genes. Transcription of viral genes is tightly regulated and acts as the main switch on ASFV gene expression in coordination with DNA replication. In total, 20 genes are currently considered to be involved in the transcription and modification of mRNAs, comprising approximately 20% of the ASFV genome. Four classes of mRNAs have been identified by their distinctive accumulation kinetics: immediate early and early genes, expressed before the onset of DNA replication, and intermediate and late genes, expressed after [24]. This transcriptional machinery gives ASFV precise configurational and temporal control of gene expression and considerable independence from its host.

Despite having a relatively low overall genomic mutation rate, the evolutionary rate of the ASFV variable regions seems to approach those of RNA viruses and can greatly affect ASFV genome structure [25,26]. ASFV strains showing MGFs gene duplication are often associated with a more violent phenotype while attenuating loss of MGFs occurs after viral passage in in vitro cell culture [22,26,27]. Comparative genomic analyses have also identified a range of genes in the constant region undergoing positive selection (e.g., CD2v/EP402R and C-type lectin/EP153R) that represent another source of genetic diversity among ASFV isolates [5,26,28]. These evolutionary processes are of considerable interest as they are key drivers of change in specific genes and encoded antigens, arguably influencing vaccination strategies and/or the stability of live attenuated vaccines, as well as diversity in host response.

### 2.3. ASFV Structural Proteins and Proteins Involved in Assembly

Up to 70 structural proteins have been identified from the ASFV virion, 16 of which, at least, are thought to be involved in assembly of the virus particle [29] (Table 1). These include the major capsid protein p72 (ORF B646L) which is essential for assembly of the icosahedral capsid on the inner envelope [30]; the mature proteins derived from polyprotein pp220 (CP2474L) and polyprotein pp62 (CP530R) who assemble to form the core shell that surrounds the DNA-containing nucleoid [31]; the membrane protein p17 (D117L), required for assembly of the capsid layer on the inner envelope [32]; the phosphoprotein p14.5 (E120R) a capsid component which mediates intracellular virus transport [33]; the enzyme responsible for polyprotein processing, encoded by ORF S273R (cysteine proteinase); and the major DNA-binding proteins p10 (K78R) and pA104R which are located in the nucleoid of mature ASFV particles [31], consistent with a role in this viral domain. pA104R, specifically, has, on the basis of knockdown experiments with small interfering RNAs [34], been shown to be involved in viral transcription, DNA replication, and genome packaging.

Although not detected in the ASFV proteome, a packaging A32L ATPase (B354L), which has orthologs in all NCLDVs has been predicted in ASFV [35], as well as a lambda-like recombinase (D345L) [17], which might be involved in processing DNA ends for strand exchange or single-strand annealing during recombination. The packaging ATPase of Mimivirus is also absent from proteomic analysis as, it has been suggested, it leaves the nonvertex packaging site after packaging and is probably reused [16]. These annotated protein sequences have yet to be functionally and biochemically characterized. The ASFV type II topoisomerase (P1192R) [36,37] is likewise absent from the ASFV proteome, but has been detected at intermediate and late phases of infection in the cytoplasm of infected cells, accumulating in viral factories [38] and, it has been argued, may participate in genome segregation, by facilitating the separation of newly-replicated DNA molecules, as suggested for Mimivirus.

**Table 1:** African swine fever virus (ASFV) Structural proteins and proteins involved in assembly.

ORF	Description	Localization	Reference(s)
B646L	Major capsid protein p72	capsid	[30,39]
B438L	Protein p49	capsid	[40]
E120R	Protein p14.5	capsid	[33]
D117L	Major transmembrane protein p17	inner envelope	[32,41]
E183L	Transmembrane protein pE183L	inner envelope	[42,43]
CP2475L	Protein p5, polyprotein pp220 derived	core shell	[44]
CP2475L	Protein p14, polyprotein pp220-derived	core shell	[31,44,45,46]
CP2475L	Protein p34, polyprotein pp220-derived	core shell	[31,44,45,46]
CP2475L	Protein p37, polyprotein pp220-derived	core shell	[31,44,45,46]
CP2475L	Protein p150, polyprotein pp220-derived	core shell	[31,44,45,46]
CP530R	Protein p8, polyprotein pp62-derived	core shell ^1^	[47]
CP530R	Protein p15, polyprotein pp62-derived	core shell	[31,45,48]
CP530R	Protein p35, polyprotein pp62-derived	core shell	[31,45,48]
S273R	Polyprotein processing protease	core shell	[49,50]
A104R	Histone-like DNA-binding protein	nucleoid	[34,44]
K78R	DNA-binding protein p10	nucleoid	[51]
P1192R	Topoisomerase II	ND	[36,37]
B354L	A32L ATPase ^2^	ND	[35]
D345L	Lambda-like recombinase ^2^	ND	[17]

^1^ subviral localization inferred from their known or predicted role. ^2^ hypotheticals. ND—not determined

## 3. The DNA Binding Protein pA104R 

### 3.1. Structure of pA104R

ASFV ORF A104R (5-AR) predicts a type II DNA-binding protein of 104 amino acid residues with a molecular weight of 11.6 kDa and a basic isoelectric point of 11 [52], which is most similar to bacterial HU (histone-like protein from *E. coli* strain U93) and Integration host factor (IHF). 

HU and IHF are the primary DNA-packaging proteins in prokaryotes, analogous in function to eukaryote histones, with which they share some characteristics, i.e., small size, basic pI, an amino acid composition rich in lysine and alanine residues and a capacity to supercoil DNA [7,53]. HU is a dimeric nucleoid-associated protein and the most abundant DNA-binding protein of E. coli that resembles the eukaryotic Histone H2B; it plays a crucial role in DNA bending and compaction and is also involved in various DNA transaction processes like replication, DNA recombination and repair, cell division, and functional interaction with specialized proteins like topoisomerase [54]. HU also influences host cell interactions and can impair the host immune response [55,56]. It exists as a homodimer in most bacteria, but as a heterodimer in *Enterobacteriaceae* whereas IHF is an obligate heterodimer. Although IHF shares both structural and sequence similarity to HU its DNA binding and bending features are strikingly different. HU binds to DNA in a random manner, while IHF is moderately sequence specific and binds tightly to its cognate recognition sites [54]. Both proteins consist of three alpha helices (the “body”) and five beta sheets (“arms”), with the beta sheets from each protomer forming a DNA binding cradle while the alpha helices form a dimerization core [54]. The beta-sheet arms can induce and/or stabilize DNA bending where conserved proline residues at the tips intercalate between base pairs in the DNA minor groove [7,57].

**Figure 1:**
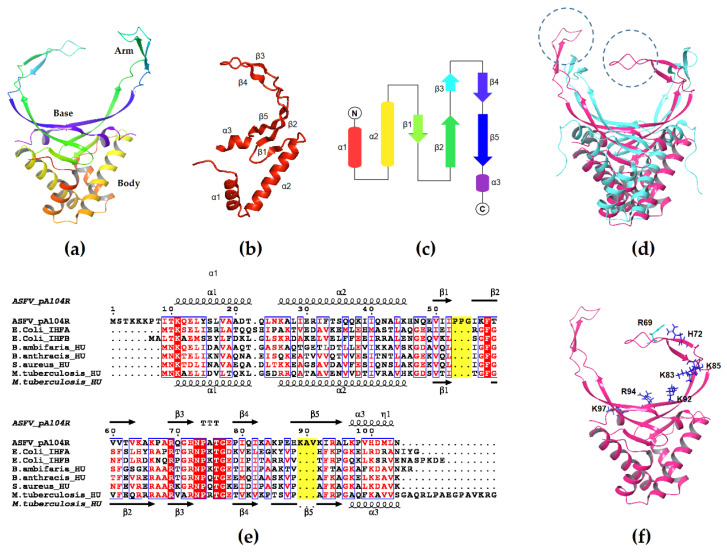
The structure of pA104R: (**a**) Model of dimeric pA104R generated by the Schrödinger Maestro suite [59]; (**b**) One protomer of the dimeric pA104R; (**c**) Topological diagram of secondary structural elements of the pA104R protomer; (**d**) Model of pA104R (magenta) superimposed with M. tuberculosis HU (cyan). The lengthened beta 2–3 and 4–5 loops are encircled; (**e**) Multiple sequence alignment of pA104R with other HU/IHF members generated by Clustal Omega (version 1.2.4) [60] using the HHalign algorithm and its default settings as described in [61] and ESPript 3.x [62]. The extra two sets of amino acids are highlighted in yellow; (**f**) The positively charged amino acid residues in the bottom (K92, R94, and K97) and arm regions (R69, H72, K83, and K85) of the pA104R protomer that contribute the majority of the protein-DNA interactions. The PDB codes of the structures are 6LMH (pA104R) and 4PT4 (mtbHU). Accession numbers are P68742, P0A6X7, P0A6Y1, B1YQ53, A0A2A7DF13, Q99U17, and P9WMK7.

The secondary structure of pA104R is highly similar to bacterial HU/IHF. It exists as a homodimer consisting of a largely alpha-helical body (alpha-helices 1, 2 and 3) capped by a DNA binding cradle formed by an antiparallel beta-sheet “bottom” (beta strands 1, 2 and 5) that extends into two arms (beta strands 3 and 4) [58] (Figure 1a–c). Unlike bacterial HU/IHF, however, the arms of pA104R appear to be lengthened by two sets of extra amino acids (residues P53 to G55 and A90 to K92), which extend the length of the beta 2–3 and beta 4–5 loops [58] (Figure 1d,e). Another notable difference is that the proline residue at the tip of the beta-sheet arms intercalates between base pairs in the major groove instead of the minor groove and does not seem to induce a DNA bend [58]. The negatively charged DNA backbone interacts with positively charged amino acid residues densely distributed in the cradle region—the bottom region of one protomer contacts the minor groove while the arm of the other protomer curls around the major groove. Three positively charged residues in the bottom region (K92, R94, and K97) and four in the arm (R69, H72, K83, and K85) seem to contribute the majority of the DNA-binding interactions [34,58] (Figure 1f). pA104R has a binding site of around 14 to 16 nt and a minimal binding length of 11 to 20 nt, which remains stable under different experimental conditions [34]. While capable of binding both ssDNA and dsDNA, binding affinity is higher with dsDNA [34,58], implying that pA104R may be more likely to compact the dsDNA genome of ASFV rather than other ssDNAs present in the cytoplasm of infected cells.

### 3.2. Role of pA104R in Viral Packaging

Some DNA and RNA viruses make use of positively charged proteins to bind and condense the negatively charged genome in an energy-independent manner [16]. Notably, Simian virus 40 uses histone proteins from the host to form minichromosomes in a nucleosome-like structure that is similar to eukaryotic chromosome architecture [63,64]. Frouco et al. show that the positively charged pA104R similarly binds DNA in an ATP-independent manner and additionally collaborates with the putative topoisomerase II identified in ASFV (P1192R) to induce DNA supercoiling [34], which, the authors speculate, may lead to the condensation of the virus pronucleoid. This same mechanism has been described in prokaryotes, where HU cooperates with topoisomerase I to bind DNA and introduce negative supercoiling, condensing DNA into nucleosome-like structures, which is one way prokaryotes are able to package their DNA into the nucleoid [65]. The idea that pA104R is profoundly involved in spatial genome organization and packaging is further supported by the observation that it is an intermediate to late phase protein which is recruited to virus factories (where virus morphogenesis and the main late phase of DNA replication occur) at later stages during virus assembly [34], an event consistent with a role in nucleoid formation. 

The same study shows that pA104R’s DNA-binding activity is maintained over a wide range of temperatures (4 to 37 ℃) and pH values, but seems to be affected by NaCl concentration, being undetectable under high-ionic-strength conditions [34], which, it is suggested, indicates that ion pair formation is involved in protein-DNA interaction as reported for other viral DNA-binding proteins involved in DNA packaging [66]. Mutagenesis studies by Frouco et al. and Liu et al. further show that binding activity is not affected when positively charged residues in the DNA-binding region of pA104R are replaced by other positively charged residues, indicating that charge is critical for protein-DNA interactions [34,58]. Involvement of positively charged domains in genome packaging has been experimentally demonstrated in many virus families [67], most relevantly in *Leviviridae* bacteriophages, which depend on an RNA binding protein A for genome packaging; the most positively charged segment of the protein A is located on the internal beta-strand in close contact with the packaged RNA, similarly to what has been described for pA104R, and mutations in positively charged amino acid residues of this domain also interfere with the viruses’ RNA packaging capacity [68]. All these facts suggest that ASFV viral DNA packaging is a highly dynamic process, and the virus may employ a hybrid mechanism with pA104R at its center for a prompt DNA compaction that may be critical to generate the high number of ASFV particles necessary to maintain a successful infection.

### 3.3. Phylogenetic Analysis of pA104R

Viral DNA-packaging proteins sometimes belong to the same family as those used by the hosts. The histones of baculoviruses (Nudivirus lineage *Heliothis zea virus* 1 H3/H4 fusion protein) and of Polydnaviruses (H4 of the bracovirus lineage), for example, are analogous to the corresponding insect histones [9]; similarly, among the NCLDVs, the genes for histones H2A, H2B, and H3 of *Marseillevirus* and *Lausannevirus*, and for all *Medusavirus* histones (H1, H2A, H2B, H3, and H4) are phylogenetically placed at the root of the eukaryotic clades [14], suggesting that they are recent derivations from their hosts. In other NCLDVs, however, viral DNA-packaging proteins are unrelated to those of the host, appearing to derive from an entirely different domain; an example is the A437L protein in *Chlorovirus* (of the phycodnavirus lineage) which is homologous to the archaeal-type MC1 chromosomal proteins [17]. Although this type of horizontal gene transfer seems more common in the Phycodnavirus-Mimivirus clade (not surprisingly, considering the greater abundance of phagocytosed and endosymbiotic bacteria in the protist hosts of these viruses), a significant number of transfers from bacteria have also substantially affected the evolution of the animal NCLDVs [17]. ASFV, specifically, appears to have acquired a discrete array of bacterial genes from prokaryotic endosymbionts or parasites of its *Suidae* hosts, such as an EF-G-like GTPase (CP312R), a NifS-like pyridoxal-phosphate-dependent enzyme (QP383R) and a distinctive HU/IHF-like prokaryotic DNA-packaging protein (A104R) [17,52]. 

**Figure 2:**
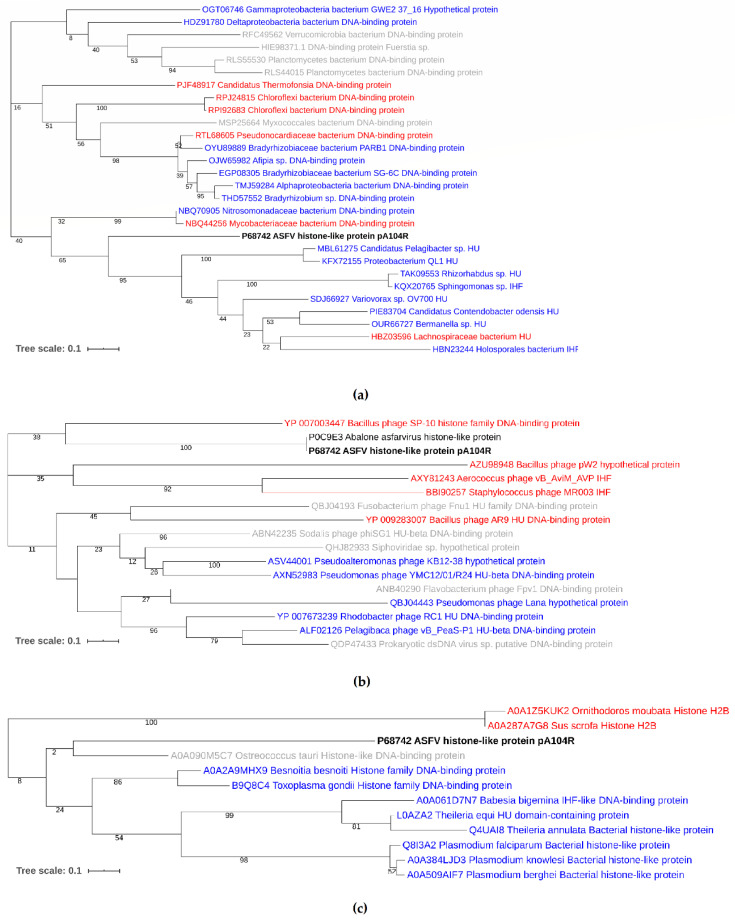
Phylogenetic analysis of pA104R: (**a**) Phylogenetic tree of DNABII bacterial sequences. Blue labels represent Proteobacteria sequences. Red labels represent Terrabacteria group sequences. Grey labels represent other bacterial sequences; (**b**) Phylogenetic tree of DNABII viral sequences. Blue labels represent sequences whose host species belong to the Proteobacteria phylum. Red labels represent sequences whose host species belong to the Terrabacteria group; (**c**) Phylogenetic tree of DNABII eukaryotic sequences. Blue labels represent organisms of the phylum Apicomplexa. *Sus scrofa* and *Ornithodoros moubata* histones H2B were chosen as an outgroup (red). A BLAST (version 2.10.0) search of the nonredundant protein database was performed using BLOSUM62 matrix and default settings (Expectation value 10, Low complexity filtering, Max No. of answers 25, Word size 3). Sequences having scores >45, E value <0.1, and >25% identity were selected. Trees were generated by MEGA X (version 10.1.8) [75] using the Maximum Likelihood method and Tamura-Nei model [76] and iTOL (version 4) [77] from a multiple sequence alignment of the relevant sequences generated by MAFFT (version 7) online service using the default settings as described in [78]. The reliability of the tree topology was assessed using Felsenstein’s bootstrap test [79], with 1000 replications. Bootstrap values are shown next to the branches. The scale bars indicate the expected number of amino acid substitutions per site. Accession numbers are indicated in the labels.

**Figure 3:**
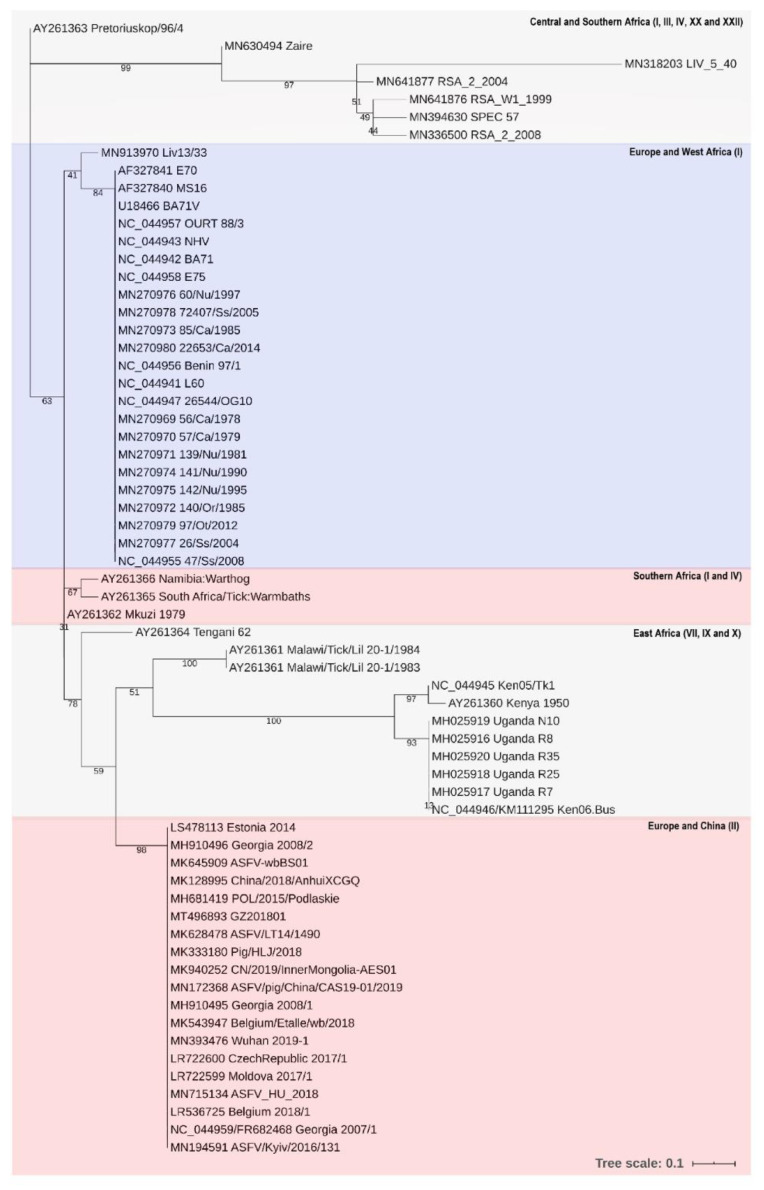
Phylogenetic tree of 64 different ASFV isolates. Geographic distribution and p72 genotype of ASFV isolates are indicated. Nucleotide sequences were obtained from NCBI nucleotide collection database. Trees were generated by MEGA X (version 10.1.8) [75] using the Maximum Likelihood method and Tamura-Nei model [76] and iTOL (version 4) [77] from a multiple sequence alignment generated by MAFFT (version 7) online service using the default settings as described in [78]. The reliability of the tree topology was assessed using Felsenstein’s bootstrap test [79], with 1000 replications. Bootstrap values are shown next to the branches. The scale bars indicate the expected number of amino acid substitutions per site. Accession numbers are indicated in the labels.

A BLAST search of the nonredundant protein database showed that pA104R shares a ∼30–40% sequence identity with DNABII homologs, being more similar to the HU family DNA-binding proteins of *Nitrosomonadaceae bacterium* (score = 72, E-value = 4 × 10^−19^, 42.11%) and *Mycobacteriaceae bacterium* (score = 70.9, E-value = 1 × 10^−18^, 41.05%) and less similar to the IHF, e.g., *Planctomycetaceae bacterium* (score = 60.8, E-value = 2 × 10^−14^, 38.04% identity) [69]. Phylogenetic tree reconstruction showed that pA104R clusters with Proteobacterial and Terrabacteria group (Actinobacteria, Firmicutes, Chloroflexi) DNA-binding proteins (Figure 2a), suggesting it might have originated from them. Interestingly, phylogenetic analysis indicates that the endosymbionts of several tick species, including the African soft tick *Ornithodoros moubata*, a natural reservoir and vector of African swine fever virus, belong to the same alpha and gamma subgroups of proteobacteria [70]. In addition, pA104R also shows substantial similarity to *Abalone Asfarvirus* A104R homolog protein (score = 189, E-value = 5.5 × 10^−18^, 41.7% identity) and with bacteriophage coded HU homologs, e.g., *Rhodobacter phage* (score = 42, E-value = 1 × 10^−5^, 34.2% identity) which infects Alphaproteobacteria, clustering with the DNA-binding protein of *Bacillus phage SP-10* (score = 30.8, E-value = 2 × 10^−2^, 36.84% identity), whose host species belong to the Firmicutes group of bacteria [69] (Figure 2b). The higher difference between pA104R and bacteriophage HUs, denoted by the longer branch lengths, may represent a case of convergent evolution. HU-like proteins are also present in eukaryotic organisms with an apicoplast, which shares sequence similarity to the secondary plastid of the closely related dinoflagellate algae [71,72]. In tick-transmitted eukaryotic pathogens of the phylum *Apicomplexa*, like *Babesia* spp., plastids contain circular DNA that is organized by HU homologs and are essential for the organisms’ survival [73]. Although supported by a low bootstrap value, most likely due to the absence of intermediate taxa and different rates of evolution, pA104R shows a vague relationship to the HU proteins of apicomplexan eukaryotic parasites, e.g., *Toxoplasma gondii* (score = 36.6, E-value = 0.029, 27.6% identity) and also, *Ostrococcus tauri*, a unicellular species of marine green alga, although the latter is most likely an artifact of tree reconstruction [69] (Figure 2c). Finally, among different virulent and non-virulent isolates, phylogenetic analysis of 64 different ASFV sequences compiled from GenBank, EMBL, DDBJ, PDB, and RefSeq revealed that pA104R is highly conserved, showing a small degree of variation consistent with p72 genotype [74] and geographic region [69]. The isolates were separated into five clusters, with two main clusters—genotype I, consisting of 22 isolates from Southern Europe and the *Benin97/1* from West Africa, and genotype II, consisting of 11 isolates from Central and Eastern Europe and 8 from Asia—and three smaller clusters of isolates from Africa (Figure 3). 

### 3.4. Potential of pA104R as a Target for Vaccine and Drug Development

Classical approaches to ASFV vaccine development have proven largely unsuccessful; inactivated viruses, while efficient at inducing antibodies which on occasion are capable of blocking virus in fluids, do not induce the specific cytotoxic cellular immune response necessary for the elimination of virus-infected cells, and the use of live attenuated viruses generated by passage in tissue culture had a poor safety profile [80,81,82]. Current approaches concentrate on the development of modified live viruses by targeted deletion of structural genes and genes involved in manipulation and evasion of host defenses or subunit vaccines. The first approach has generated live attenuated viruses that confer a level of protection against infection [83,84,85,86], but need further improvement, mainly from a biosafety perspective [87] since on most occasions protection against the parental virulent strain (homologous virus) is limited [82]. The latter approach is safer and has the added advantage of being a differentiation of vaccination from infected animals (DIVA) compliant, but information on which viral antigens to include is lacking. Screening of potential ASFV antigenic determinants capable of inducing antibody responses after immunization in vivo has been challenging [88,89,90] due, partly, to the virus’s complex nature; nevertheless, several have been recently identified which, when pooled together, are capable of inducing protection against lethal challenge with a virulent strain [91]. pA104R is one of 12 viral proteins previously demonstrated to be the main targets of serological immunity in pigs, and the antibody response to pA104R is higher in asymptomatic than in chronically infected animals, which, it is suggested, indicates that antibodies against this protein might be an indicator of an effective immune response or that this response is somehow involved in protection [52,92]. To our knowledge, however, pA104R has never been included in subunit vaccine formulations, thus its protective efficacy needs to be confirmed. An alternative approach that attempts to bridge the gap between gene deletion and subunit vaccines relies on the generation of recombinant viruses with limited replication capacity. These viruses, it is theorized, are incapable of producing infectious virions, yet they can still elicit an immune response in the host [93,94,95,96]. Deletion of the pA104R gene from the non-pathogenic Ba71V by Freitas et al. yielded a replication defective DISC mutant (Ba71V∆pA104R) which unfortunately could not be isolated [97]. The authors speculate that very low expression of pA104R in the complementary cell line may not have allowed replication of the recombinant virus. Development of a more stable cell line and other strategies are being explored. 

In the absence of a vaccine, antivirals can be used to reduce the number of susceptible animals and control the spread of infection. Although a number of promising anti-ASFV agents have been described (e.g., nucleoside analogs [98], interferons [99], antibiotics [100], and small interfering RNAs [101]), their high cost often precludes evaluation of in vivo efficacy. Compounds such as the stilbenes resveratrol and oxyresveratrol [102], polyphenolic phytoalexins produced by grapevines and many other plants, thus stand out, as they can be extracted from natural sources. Stilbenes are one of the two regioisomers of the diarylethene class of compounds that occur naturally in different botanical families, and their antiviral action against various families of DNA and RNA viruses is well documented [103,104,105]. Rather than solely targeting one viral protein, they seem to also regulate molecular pathways involved in the control of virus infection and act as immune adjuvants [106]. This fact presents itself as an additional advantage, considering resistance to drugs that only target viral proteins frequently appears as the viruses mutate. A wide range of molecular targets for the stilbene class of compounds have been identified, including the type II eukaryotic topoisomerase [107] and, most relevantly, the HU protein of M. tuberculosis [108]. The same two compounds that have demonstrated activity against the M. tuberculosis HU were found to likewise inhibit the pA104R-DNA interactions [35]. It is thus tempting to speculate that the inhibitory effect of resveratol and oxyresveratol on ASFV DNA replication, late viral synthesis, and factory formation, might also be due, in part, to pA104R-targeting. The most promising of the two compounds, SD4 (MtbHU-in-1), showed low cytotoxicity and reduced ASFV replication in a dose dependent manner, which, the authors argue, further confirms the vital role of pA104R in the ASFV replication cycle and highlights its potential as a target for antiviral therapy. Several other compounds from this class have demonstrated antimycobacterial properties [109,110,111] but have yet to be tested against ASFV.

## 4. Conclusions

The mechanism of genome encapsidation in ASFV has not been fully characterized, but evidence suggests it shares similarities to that of Mimivirus and other large NCLDVs, which rely on a packaging ATPase, a putative type II topoisomerase, recombinase/s, and possibly several as yet unidentified components that are integral parts of the prokaryotic chromosome segregation and translocation machinery. ASFV also encodes a packaging A32L ATPase (B354L), which has orthologs in all NCLDV, a lambda-like recombinase (D345L) and a type II topoisomerase (P1192R), as well as the distinctive HU/IHF-like prokaryotic DNA-packaging protein (A104R).

pA104R is highly conserved among virulent and non-virulent ASFV isolates, and phylogenetic tree reconstruction shows a high degree of similarity with Proteobacterial and Terrabacteria group DNA-binding proteins, suggesting it might have originated from them. Research indicates it may be profoundly involved in spatial genome organization and packaging, collaborating with topoisomerase II to induce DNA supercoiling, potentially leading to the condensation of the virus pronucleoid prior to docking, a mechanism that has been described in prokaryote genome packaging. In theory, interfering with this mechanism may result in disruption of viral infection i.e., in the generation of viral particles devoid of viral DNA or with unusual DNA structures, while still being capable of inducing the immune response required for adequate immunization of the host.

Current approaches to the ASFV vaccine design concentrate on the development of modified live viruses by targeted gene deletion or subunit vaccines, with mixed results. For an effective intervention against African Swine Fever, targets should have an essential role in the ASFV replication cycle, and be genetically stable, as the virus will have less opportunity to escape without phenotypic costs. pA104R seems to be both. A pA104R replication defective DISC mutant (Ba71∆pA104R) has already been generated but not successfully isolated. Efforts are being made into the development of a more stable complementary cell line. Other strategies should be explored, which could involve including pA104R in subunit vaccine formulations, considering it is one of the main targets of serological immunity in pigs, and the antibody response against pA104R may be involved in protection. In the absence of a vaccine, pA104R presents itself as a promising target for antiviral therapy, with the stilbene class of compounds having already proved capable in reducing ASFV replication in vitro.
